# Mechanistic insights into avian and chiropteran thermal risk exposure in a warming world

**DOI:** 10.1093/conphys/coag060

**Published:** 2026-08-24

**Authors:** V M Mdluli, M J Noakes, S R Conradie

**Affiliations:** School of Animal, Plant, and Environmental Sciences, University of the Witwatersrand, Johannesburg, 1 Jan Smuts Avenue, Braamfontein, 2017, South Africa; School of Animal, Plant, and Environmental Sciences, University of the Witwatersrand, Johannesburg, 1 Jan Smuts Avenue, Braamfontein, 2017, South Africa; School of Animal, Plant, and Environmental Sciences, University of the Witwatersrand, Johannesburg, 1 Jan Smuts Avenue, Braamfontein, 2017, South Africa

**Keywords:** Biophysical ecology, dehydration, ecophysiology, sublethal fitness costs, thermal risks

## Abstract

Climate change threatens biodiversity by challenging animals’ ability to balance energy and water budgets, across timescales ranging from (i) acute-lethal (hours-to-days) to (ii) chronic-sublethal (days-to-weeks). However, predicting the effects of heat exposure over these timescales is complicated by inter- and intraspecific variability in traits, such as physiological tolerances, life-history strategies and the surrounding microhabitat. To address these challenges, we synthesize current knowledge of the physiological and behavioural mechanisms underpinning acute and chronic thermal risks in small endotherms, focusing on birds and bats. We review thermal risks and the key traits determining vulnerability from a biophysical modelling perspective. We then critically evaluate the growing use of biophysical models to predict thermal vulnerability, emphasizing their value but also the risk of oversimplification in the absence of empirical validation. Importantly, most models assume fixed physiological trait values and thermal thresholds and do not incorporate phenotypic plasticity. We also identify key knowledge gaps that constrain predictive capacity, including limited data on dehydration tolerance and heat tolerance thresholds, particularly in bats; incomplete understanding of reaction norms and acclimatory timescales; and poor resolution of how individual thermal trade-offs scale to population dynamics.

## Introduction

Anthropogenic climate change is undoubtedly one of the most pressing threats to biodiversity in the 21st century, driving widespread population declines across taxa (Welbergen *et al.,* 2008; Urban, 2015; Conradie *et al.,* 2019; Pattinson *et al.,* 2022). Rising air temperatures (*T*_air_) directly affect animals, which over time can lead to adaptive responses in their morphology, physiology and behaviour (Boyles *et al.,* 2011; Gardner *et al.,* 2019) and restructuring or even breakdown of communities (Riddell *et al.,* 2019). However, understanding and predicting the extent to which anticipated climate change will affect species is complicated by inter- and intraspecific variability in traits, such as physiological tolerances (Oswald & Arnold, 2012; Noakes *et al.,* 2016), life-history strategies (Jenouvrier, 2013) and the surrounding microhabitat (Strydom *et al.,* 2024).

Most large-scale thermal vulnerability frameworks either rely on correlative associations that ignore mechanism or assume fixed physiological trait values over space and time (Erasmus *et al.,* 2002; Briscoe *et al.,* 2023). While these are useful for identifying broad patterns of risk, they fail to capture adaptive physiological responses as a potential buffer. Biophysical modelling, based on first principles of thermodynamics to simulate energy, mass and water fluxes in response to environmental conditions (Porter & Gates, 1969; Bakken, 1976; Kearney & Porter, 2009) provides an opportunity to overcome these challenges and provide robust predictions of the impacts of climate change (Briscoe *et al.,* 2023), identify at-risk populations (Zhang *et al.,* 2025) and develop conservation interventions (Zhong *et al*., 2025). Biophysical models have been used to predict the effects of climate change on birds (Kearney *et al.,* 2016; Riddell *et al.,* 2021; Conradie *et al.,* 2024), revealing that the extent and magnitude of thermal risk exposure costs can vary among regions and species. However, these models are limited by the paucity of knowledge on thermal physiology for some taxa and typically assume fixed trait values, ignoring phenotypic plasticity. Accurate predictions require a mechanistic understanding of thermoregulatory responses and incorporation of adaptive capacity, if they are to be used for conservation planning and action.

This review synthesizes our current understanding of the mechanisms underlying acute and chronic thermal risks posed to small endotherms, specifically birds and bats, from a biophysical perspective. We focus on these two groups due to their shared traits, physiological responses and vulnerability to extreme environmental conditions, as evidenced by mass mortality events reported for both groups at the same location, on the same day (McKechnie *et al.,* 2021*a*). We do this by reviewing what we know about thermal risks across a temporal continuum, from acute, lethal (hours-to-days) to chronic, sublethal (days-to-weeks) and across an environmental gradient, from dry to humid. We outline the shared and unique traits that make birds and bats vulnerable to climate change and consider the potential for these small endotherms to undergo adaptive responses. We finally critically examine the strengths and limitations of biophysical models to understand and predict these responses and highlight important questions going forward if we aim to inform and guide conservation strategies for species currently at risk and those predicted to be in the future.

## Thermal risks of heat

**Figure 1:**
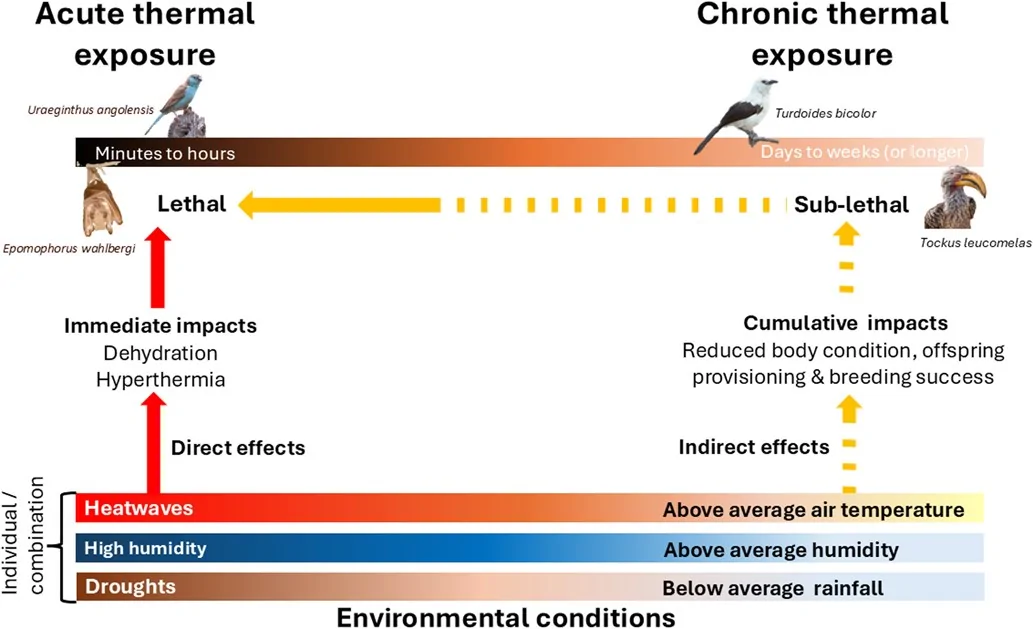
Thermal risk exposure for small endotherms ranges from acute, direct effects to chronic, indirect effects depending on the environmental conditions experienced. Acute thermal exposure, occurring over minutes to hours during extreme events, such as heatwaves, high humidity and droughts, can result in direct lethal consequences such as dehydration and hyperthermia. Chronic thermal exposure, occurring over days to weeks (or longer) during persistently elevated environmental conditions, can lead to indirect sub-lethal effects such as reduced body condition, decreased offspring provisioning and lowered breeding success. Over time, these sublethal effects can compound and result in lethal consequences.

Small endotherms such as birds and bats are among the most vulnerable to high environmental heat load and the adverse effects of climate change (Welbergen *et al.,* 2008; McKechnie & Wolf, 2010; Albright *et al.,* 2017; Ratnayake *et al.,* 2019). Due to their small body sizes and the energetic demands of flight, birds and bats tend to have high mass-specific metabolic rates and high surface-area-to-volume ratios resulting in lower thermal inertias (Weathers, 1981; Canals, 1998). Coupled with these demands, a small body size limits the capacity for absolute energy and water storage (although small-bodied animals can accumulate substantial energy reserves over short periods, i.e. migratory fattening), highlighting the importance of energy and water conservation strategies, particularly in the heat. To withstand high operative environmental temperature [combined effects of *T*_air_ (*T*_e_), solar radiation, longwave radiation, conductance and convection, providing a measure of the net environmental heat load that an animal experiences in a given microclimate]_,_ small endotherms may adjust their behaviour {e.g. shade-seeking, activity levels (Cunningham *et al.,* 2021) and physiology [e.g. increased evaporative water loss (EWL), facultative hyperthermia; Dawson, 1954*a*; McKechnie *et al.,* 2021*a*]}. Acute exposure to high *T*_e_ may result in lethal hyperthermia or dehydration while prolonged exposure to above-average *T*_e_ may result in missed opportunities leading to sublethal fitness costs (du Plessis *et al.,* 2012; van de Ven *et al.,* 2019) or even somatic damage (Lim, 2018; Nord *et al.,* 2026). Thus, the consequences of thermal exposure occur across a temporal continuum, ranging from acute, lethal (short-term, typically minutes-to-hours) to chronic, sublethal (prolonged, typically days-to-weeks or longer) heat exposure (Fig. 1). In the section that follows, we review the mechanistic links and traits that underlie species vulnerability to heat along this temporal continuum and the conservation implications.

### Acute exposure to heat

When *T*_e_ > body temperature (*T*_b_) , passive heat dissipation is no longer possible, and EWL becomes the primary avenue of heat dissipation for endotherms to maintain *T*_b_ below lethal limits (Salt, 1964; McKechnie & Wolf, 2019). Acute heat exposure can result in the need to balance increasing EWL to avoid lethal hyperthermia and conserving water to avoid lethal dehydration (Fig. 1; McKechnie & Wolf, 2019). This need to balance heat dissipation and water conservation is exacerbated in birds and bats, as they have larger relative surface areas for heat gain and a lower capacity to store water compared to larger animals (Weathers, 1981; Canals, 1998).

In recent decades, increases in the frequency and intensity of extreme heat waves have led to multiple mass mortality events of birds and bats (Welbergen *et al.,* 2008; Ratnayake *et al.,* 2019; McKechnie *et al.,* 2021*b*). For instance, ~45 500 flying foxes (*Pteropus* species) died when *T*_air_ exceeded 42°C during a single day in south-east Queensland, Australia (Welbergen *et al.,* 2014), and ~23 000 spectacled flying foxes (*Pteropus conspicillatus*) died during a 2-day heat wave in northern Australia, nearly one-third of the estimated total national population (Mao, 2019). Mass mortality events have also been reported in Australian birds (McKechnie *et al.,* 2012), including thousands of zebra finches (*Taeniopygia guttata*) and budgerigars (*Melopsittacus undulatus*) when maximum *T*_air_ exceeded 45°C for consecutive days (Towie, 2009; McKechnie *et al.,* 2012) and 208 endangered Carnaby’s black-cockatoos (*Calyptorhynchus latirostris*) during a single day in 2010 when *T*_air_ exceeded 47°C (Towie, 2010; Saunders *et al.,* 2011). While less well documented, heat-related mortality events have been observed in other regions, including insectivorous bats in Italy (Russo *et al.,* 2025) and Cambodia (Pruvot *et al.,* 2019), and birds (mostly passerines) and fruit bats in southern Africa (McKechnie *et al.,* 2021*b*). The underlying cause of these heat-related mortalities has been attributed to lethal hyperthermia (Welbergen *et al.,* 2008; Mo *et al.,* 2022), dehydration (Festa *et al.,* 2023) or a combination of the two (McKechnie & Wolf, 2010; Wildlife Health Australia, 2025), but empirical data illustrating relative contributions of these responses are limited (McKechnie *et al.,* 2012). There are also reports of heat-related mortalities in juvenile birds and bats during heat waves, which could result from lethal dehydration, hyperthermia or roost abandonment (McCowan & Griffith, 2021; Mo *et al.,* 2022; Sloane *et al.,* 2022; Russo *et al.,* 2025). The proximate cause of heat-related mortality likely varies among taxa and regions, and a better understanding of dehydration tolerance limits and lethal hyperthermia thresholds in different species is needed to resolve this uncertainty. Understanding these drivers can help identify conservation interventions such as water provisioning, artificial shading and protection of thermal refugia.

Lethal dehydration in the heat occurs when the rate or magnitude of water loss exceeds an animal’s capacity to maintain hydration. However, tolerance limits of dehydration remain poorly studied in birds and bats (Studier, 1970; Arad *et al.,* 1989*a*; Wolf &Walsberg, 1996). Wolf and Walsberg (1996) reported that free-ranging verdins (*Auriparus flaviceps*; body mass ≈ 7 g) in Arizona experienced *T*_air_ ≈ 55°C in the full sun at 12:00 on a summer day, which would require them to lose ~7% of their body mass per hour via EWL. These authors suggest the dehydration tolerance limit of verdins is ~11% of body mass citing unpublished data, meaning that verdins foraging in the above scenario could be at risk of lethal dehydration within 1.6 h (Wolf & Walsberg, 1996; Wolf, 2000). In contrast, rock pigeons (*Columba livia*) survived losing 16–18% of body mass when water-deprived for 48 h at *T*_air_ ≈ 50°C, recovering 97% of their body mass within 30 min of drinking (Arad *et al.,* 1989*a*). This suggests that dehydration tolerance limits may be higher in non-passerine than passerine birds, possibly due to taxonomic differences in avenues of EWL at high *T*_e_. Respiratory EWL via panting is the primary avenue of heat loss at high *T*_e_ for passerine birds, which require increases in ventilation and metabolic rate, increasing internal heat loads to dissipate heat (Wolf & Walsberg, 1996; Tieleman & Williams, 2002; McKechnie *et al.,* 2017). In contrast, many non-passerine species increase cutaneous EWL or respiratory EWL via gular fluttering at high *T*_e_, which requires shallower increases in metabolic rate than panting and is thus more economical in energy and water use (Smith *et al.,* 2015; McKechnie *et al.,* 2016; Talbot *et al.,* 2017). Recent studies using biophysical models to predict the risk of lethal dehydration in small passerine birds under current and future climates have taken a conservative approach, assuming a dehydration tolerance limit of 15% of body mass loss within 5 h (Albright *et al.,* 2017; Conradie *et al.,* 2019, 2020). We are only aware of one study that has investigated the dehydration tolerance limits for bats, which reports that a diurnal body mass loss of 22.8–32.3% was associated with ~50% mortality rates in four *Myotis* species (Studier, 1970). However, these measurements were taken at roost sites at moderate *T*_air_ (consistently <32°C; Studier, 1970) and may not reflect dehydration tolerance limits during extreme heat exposure when EWL rates will be substantially higher. More work is needed to quantify the dehydration tolerance limits of birds and bats, which is crucial to understand and identify their thermal risk at high *T*_e_.

Lethal hyperthermia occurs when an animal experiences excessive heat gains relative to heat dissipation, leading to *T*_b_ rising above tolerable levels. Evaporative cooling capacity refers to the total amount of heat an animal can dissipate and is often measured as evaporative scope (i.e. magnitude they can increase EWL above basal levels during acute heat exposure; Dawson, 1982; Czenze *et al.,* 2020*a*). Evaporative cooling efficiency is also important at high *T*_e_ and is typically measured as the ratio between evaporative heat loss and metabolic heat production at a given *T*_e_ (Lasiewski *et al.,* 1966; Licht & Leitner, 1967; Noakes *et al.,* 2016). A review reported maximum measured *T*_b_ of 44–47°C in birds, ~45°C in small insectivorous bats and 37–40°C in fruit bats (McKechnie & Wolf, 2019) but highlighted that comparisons of maximum *T*_b_ among taxa are limited due to varying experimental approaches and *T*_e_ ranges of measurements (McKechnie & Wolf, 2019; Noakes *et al.,* 2021). Whitfield *et al*. (2015) developed a protocol to quantify heat tolerance and evaporative cooling capacity in small endotherms by increasing *T*_e_ in a stepwise fashion until individuals show physiological (rapid changes in *T*_b_, EWL or resting metabolic rate) or behavioural signs (sudden frantic activity or loss of coordination or balance) of approaching severe hyperthermia (Whitfield *et al.*, 2015). This approach yields comparable data on heat tolerance limits (maximum *T*_air_ reached), maximum *T*_b_ and evaporative cooling capacity and efficiency, and results approximate measurements under steady-state conditions (Short *et al.,* 2022). This protocol has been widely used in birds, leading to reviews and meta-analyses (e.g. Gerson *et al.,* 2019; McKechnie & Wolf, 2019; McKechnie *et al.,* 2021*a*; Freeman *et al.,* 2022) and more recently in bats (Czenze *et al.,* 2020*a*; Noakes *et al.,* 2021; Czenze *et al.,* 2022*a*, 2024; de Mel *et al.,* 2024). Comparable data on heat tolerance and evaporative cooling capacity are crucial for understanding variation in lethal dehydration and hyperthermia risk among species under current and future climates (Albright *et al.,* 2017; Conradie *et al.,* 2024).

Some species are capable of facultative hyperthermia, tolerating an increase in *T*_b_ above normothermic levels during acute heat exposure (Tieleman & Williams, 1999*a*; Gerson *et al.,* 2019; Walker *et al.,* 2025). When *T*_b_ increases above *T*_e_, this maintains a thermal gradient for passive heat loss to the environment, reducing evaporative water costs of the individual (Weathers, 1981; Tieleman & Williams, 1999*b*; Gerson *et al.,* 2019). At extremely high *T*_e_, maintaining *T*_b_ > *T*_e_ may no longer be possible, but facultative hyperthermia can still reduce EWL demands by minimising the thermal gradient between *T*_b_ and *T*_e_ (Dawson, 1954*b*; Tieleman & Williams, 1999*a*; Gerson *et al.,* 2019). The capacity for facultative hyperthermia varies among taxa and body size, being more common in smaller birds and mammals (Gerson *et al.,* 2019; McKechnie & Wolf, 2019). The most extreme record of facultative hyperthermia use to date is from the red-billed quelea (body mass ≈ 18 g; *Quelea quelea*) in South Africa, which increased *T*_b_ to 48.0 ± 0.7°C at *T*_e_ > 45°C (Freeman *et al.,* 2020). Despite reducing EWL demands, there are risks associated with facultative hyperthermia, including that *T*_b_ is maintained at levels closer to the maximum tolerable *T*_b_ of an individual, thus decreasing the thermal safety margin if there are further increases in *T*_b_. High *T*_b_ can also cause cellular damage, including protein denaturation, reduced mitochondrial function and oxidative stress, which may lead to cell death (Iba *et al.,* 2025). Cellular damage can also lead to somatic effects, including inflammation, coagulopathy and organ damage (Aengwanich & Simaraks, 2005; McKechnie & Wolf, 2019; Iba *et al.,* 2025). How animals that tolerate extremely high *T*_b_ can avoid or mitigate somatic damage is largely unclear, but is likely facilitated by high levels of heat shock protein expression and the use of *rete opthalmicum* to maintain brain temperature below lethal limits (Freeman *et al.,* 2020, 2025).

### Chronic exposure to heat

Birds and bats commonly use behavioural thermoregulation to reduce heat loads and increase rates of heat loss when *T*_e_ exceeds the thermoneutral zone but remains below normothermic *T*_b_ (du Plessis *et al.,* 2012; Martin *et al.,* 2015; van de Ven *et al.,* 2019). In birds, such behaviours include shade-seeking, reduced daytime activity and postural changes (e.g. wing spreading). Bats display some similar behaviours, such as licking their bodies to increase cutaneous evaporative cooling and wing fanning to enhance convective cooling (Reeder & Cowles, 1951; Welbergen *et al.,* 2008; Downs *et al.,* 2015). These behaviours help reduce the risk of lethal heat exposure and/or heat load (Carroll *et al.,* 2015; Martin *et al.,* 2015) but can still impose sublethal fitness costs by reducing opportunities to engage in other important activities such as foraging, predator avoidance and offspring provisioning (van de Ven *et al.,* 2019; Sharpe *et al.,* 2021). While there is growing research quantifying the links between behavioural thermoregulation and sublethal fitness costs of birds (Sharpe *et al.,* 2019; Cunningham *et al.,* 2021), similar work on bats is lacking.

To date, most reports of sublethal fitness costs related to missed opportunities for birds have been reported in southern subtropical regions and highlight *T*_air_ > 35°C as an apparent threshold for compromised body mass maintenance (du Plessis *et al.,* 2012; Cunningham *et al.,* 2013; Wiley & Ridley, 2016; Bourne *et al.,* 2020). For instance, in southern Africa, southern pied babblers (*Turdoides bicolor*) and southern yellow-billed hornbills (*Tockus leucomelas*) have lower foraging efficiency (i.e. successful foraging returns per unit time), leading to zero daytime body mass gain on days where *T*_air_ > 35.5°C and zero 24 h body mass gain on days where *T*_air_ > 38.5°C (du Plessis *et al.,* 2012; van de Ven *et al.,* 2019). While the mass-loss thresholds for survival remain poorly defined, jacky winters (*Microeca fascinans*) in Australia experienced a 3-fold increase in adult mortality during a 2-month period with above-average *T*_air_, although the causal drivers of this pattern are likely multifaceted (Sharpe *et al.,* 2019). Individual behaviours can therefore have long-term consequences for population persistence, even when conditions do not match those associated with mortality. Moreover, the compounding effects of sublethal fitness costs may be more influential in determining population structure and persistence than direct mortality associated with acute heat exposure (Conradie *et al.,* 2019), although their relative importance will depend on factors such as population size and the severity and frequency of acute exposure events. Given the extent to which sublethal effects could restructure populations, conservation interventions should aim not only to prevent mortality but also to buffer the cumulative effects of moderate-to-hot weather.

Additionally, heat dissipation behaviours and compromised body condition may result in reduced nestling provisioning rates and nest abandonment (and thus increased nest predation (Oswald *et al.,* 2008)) and ultimately lower reproductive success (Cruz-Mcdonnell & Wolf, 2016; Wiley & Ridley, 2016; van de Ven *et al.,* 2019). Under projected future climates, sublethal fitness costs relating to breeding and nestling success are likely to become increasingly common and of major concern for conservation. For example, southern yellow-billed hornbills and southern fiscals (*Lanius collaris*) are projected to experience a 4-fold increase in the number of consecutive days where conditions are associated with delays in fledging and reductions in nestling size (up to >20 consecutive days per year), leading to higher rates of breeding failure by the end of this century (Conradie *et al.,* 2019). The impacts of these heat-related thermoregulatory trade-offs are likely to have long-term impacts across both arid and humid environments (Conradie *et al.,* 2019; Quintana *et al.,* 2022). While most documented thermoregulatory trade-offs relate to foraging and parental investment, others relating to predator avoidance and territorial defence are also important (Soravia *et al.,* 2025).

While small birds are typically diurnal, bats are active during cooler nighttime hours and are vulnerable when confined to roosts during the hottest part of the day. When bats experience high heat loads, they may respond by seeking cooler microclimates or trading off important activities such as foraging, predator avoidance or offspring provisioning for thermoregulation, leading to sublethal fitness costs (Reeder & Cowles, 1951; Welbergen *et al.,* 2008; Downs *et al.,* 2015). For instance, when *T*_air_ > 48°C, Bondarenco *et al.* (2014) reported that free-ranging little broad-nosed bats (*Scotorepens greyii*) abandoned poorly insulated roosts (e.g. small dead trees) during the day, incurring substantial metabolic costs of activity. Roost abandonment is also associated with increased predation risk (Lima & O’Keefe, 2013) and decreased reproductive success due to pup abandonment (Mo *et al.,* 2022; Russo *et al.,* 2025). Indeed, Mo *et al.* (2022) demonstrated that pup deaths during heat waves result from being abandoned by their mother, with an estimated death of ~2612 flying-fox pups during the summer of 2019–2020 in south-eastern New South Wales, Australia. Increased adult thermoregulatory behaviours during the rest phase could also lead to sleep deprivation, resulting in increased metabolic demand and EWL rates (Downs *et al.,* 2015; Roy *et al.,* 2020). The parallels between birds and bats suggest that behavioural thermoregulation imposes universal ecological trade-offs across taxa, though the degree and mechanisms may vary with diel activity patterns and microsite use. Furthermore, these examples provide some quantitative understanding of behavioural trade-offs in bats, but more empirical data are needed to elucidate the mechanisms driving sublethal fitness costs in bats (see emerging questions below).

### Interacting effects of heat and humidity

Climate change is associated not only with increases in *T*_air_ and the frequency and intensity of heatwaves but also with shifts in other climatic variables, including humidity. While climate change projections predict relative humidity to remain approximately constant, rising *T*_air_ will increase the capacity of air to hold water vapour, leading to higher absolute humidity (Willett *et al.,* 2007). Consequently, animals may experience greater evaporative cooling challenges even when relative humidity remains unchanged, because the increased water vapour content of the air reduces the gradient driving evaporative heat loss. Moreover, models predicting the impacts of climate change on animals have traditionally used projected increases in *T*_air_ (Albright *et al.,* 2017; Conradie *et al.,* 2019, 2020), and more recently *T*_e_ (Conradie *et al.,* 2024), limiting our understanding of how humidity affects thermal risk. This is largely due to our understanding of thermal responses having primarily focused on *T*_air_ alone, with comparatively fewer studies on the interacting effects of heat and humidity on thermoregulatory performance in birds (but see Lasiewski *et al.,* 1966; Gerson *et al.,* 2014; Freeman *et al.,* 2024) and bats (but see Licht & Leitner, 1967).

With increasing absolute humidity, the water vapour deficit between an individual and the surrounding air becomes smaller, reducing evaporative cooling efficiency and causing their heat tolerance limits to be reached at lower *T*_air_ (van Dyk *et al.,* 2019; Freeman *et al.,* 2024; Liddle *et al.,* 2025). Consequently, the effectiveness of heat dissipation varies across climatic regions, with arid environments facilitating greater EWL due to steeper water vapour gradients than humid habitats. For instance, the heat tolerance and evaporative cooling capacity of 30 southern African bird species varies among sites across a humidity gradient, with arid-zone birds typically having lower maximum recorded *T*_b_ and higher rates of EWL at high *T*_air_ compared to birds from more humid regions (Freeman *et al.,* 2024). Higher *T*_b_ in birds from humid environments suggests a greater capacity for facultative hyperthermia, which would decrease their reliance on evaporative cooling but reduce their thermal safety margin as *T*_b_ is maintained closer to lethal limits (Freeman *et al.,* 2024). Together, these patterns suggest that humidity may shape avian thermal physiology across different environments (Gerson *et al.,* 2014; Freeman *et al.,* 2022).

These habitat-level differences in thermal vulnerabilities indicate that the mechanisms underlying heat-related mortality risk may differ across climates, with lethal dehydration posing the greatest threat in arid environments and lethal hyperthermia in humid environments. Indeed, humid conditions are projected to result in a substantially greater risk of lethal hyperthermia compared to dry air conditions for trumpeter hornbills (*Bycanistes bucinator*) and blue waxbills (*Uraeginthus angolensis*) under anticipated climate change (Coulson *et al.,* 2025; Liddle *et al.,* 2025). The presence of this potential physiological risk gradient between arid and humid environments suggests that thermal vulnerability assessments cannot be predicted from *T*_air_ alone and that humidity may be an important proximate cause of mortality. This underscores the importance of empirical studies aimed at untangling the combined role of heat and humidity in small endotherms’ thermoregulation across heterogeneous landscapes. The outcomes of which can be used to map exposure to wet-bulb temperatures, which directly integrates *T*_air_ and humidity, to identify regions and species at risk (Coulson *et al.,* 2025).

## Key traits determining species vulnerability to heat

Thermoregulation is a response to environmental conditions (reviewed above), but these responses are also tightly linked to an individual’s morphology, physiology and life-history traits. Variation in body size and shape along climatic gradients has been extensively studied, leading to biogeographic rules such as Bergmann’s (larger body sizes in cold climates) and Allen’s (shorter appendages in cold climates) rule (Bergmann, 1847; Allen, 1907). Thus, these morphological traits are routinely incorporated in thermal vulnerability assessments. How life history and behaviour influence species responses to climate is well understood, but these relationships are far more complex, limiting their inclusion in thermal vulnerability assessments. In this section, we review how these intrinsic traits determine species’ exposure and sensitivity to high *T*_air_ and how they shape an individual’s acute lethal or chronic sublethal risks to climate change.

### Morphology

Body size and shape strongly influence heat exchange between an animal and its surrounding environment. Changes in body size in relation to climate change have been documented among several endotherms, with several bird and mammal species decreasing body mass with increasing *T*_e_ over time, consistent with Bergmann’s rule (Gardner *et al.,* 2011; Sheridan & Bickford, 2011; Ryding *et al.,* 2021). In birds, several physiological traits related to heat tolerance and evaporative cooling scale with body mass, largely attributed to the inverse relationship between surface-area-to-volume ratio and body mass (Gerson *et al.,* 2014, 2019; McKechnie *et al.,* 2021*a*). When current *T*_e_ < actual *T*_b_, a higher surface-area-to-volume ratio enables greater heat dissipation rates. In contrast, when *T*_e_ rises above *T*_b_, the thermal gradient for heat exchange is reversed, and smaller endotherms gain heat more rapidly. To offset heat gain, smaller birds and bats increase EWL more rapidly with increasing *T*_e_ > *T*_b_, increasing their risk of lethal dehydration relative to larger counterparts (Weathers, 1981; McKechnie & Wolf, 2010). Furthermore, larger endotherms tend to have lower mass-specific EWL rates and a greater absolute capacity for water storage, which may explain why heat tolerance limits scale positively with body mass among birds (McKechnie & Wolf, 2010; McKechnie *et al.,* 2021*a*). The latter pattern is somewhat counterintuitive given that higher surface-area-to-volume ratios in smaller birds could facilitate greater rates of heat dissipation, but this likely reflects the high metabolic costs of panting constraining evaporative cooling efficiency in small passerines at high *T*_e_ (Gerson *et al.,* 2019; McKechnie *et al.,* 2021*a*).

Smaller birds and bats appear to have a higher mortality risk during extreme heat events (Albright *et al.,* 2017; McKechnie *et al.,* 2021*b*; Czenze *et al.,* 2022*a*). For instance, *Pipistrellus pygmaeus* (body mass ≈ 5 g) was predicted to have daytime evaporative water costs about double those of the larger *Nyctalus noctula* (body mass ≈ 30 g), relative to their respective body mass when roosting during a heat wave in Poland (Czenze *et al.,* 2022*a*). Furthermore, ~90% of the avian mortalities during a heat wave in eastern South Africa were passerines, the majority of which were small-bodied species (McKechnie *et al.,* 2021*b*). An exception appears to be members of the family Pteropodidae (fruit bats), which include the largest bat species and have high mortality rates during extreme heat events (Welbergen *et al.,* 2008; Ratnayake *et al.,* 2019; McKechnie *et al.,* 2021*b*; Stock, 2026). The high vulnerability of fruit bat species may reflect their roosting in microsites exposed to direct solar radiation (i.e. on the foliage of trees) and their relatively low maximum tolerable *T*_b_ (~37–40°C; Welbergen *et al.,* 2008; McKechnie *et al.*, 2019; although see: Walker *et al.,* 2025). In contrast, smaller insectivorous bat species typically roost in more thermally buffered microsites (e.g. tree cavities and crevices) and tend to have higher maximum tolerable *T*_b_ ≈ 42°C (McKechnie *et al.*, 2019; Noakes *et al.,* 2021), highlighting the importance of considering ecological and physiological factors alongside morphology.

Morphological structures and appendages (e.g. bills, legs, bare skin, nose leaf, patagium) can also serve as thermal windows for heat exchange (Scholander *et al.,* 1950; Irving & Krog, 1955; Scholander, 1955). When *T*_e_ < *T*_b_, heat dissipation can be facilitated by increased blood flow to highly vascularized windows, allowing for heat loss to the environment (Arad *et al.,* 1989*b*; Tattersall *et al.,* 2009). This commonly occurs through vasomotor reactivity in endotherms, with blood flow to more poorly insulated peripheral structures increased via arteriovenous anastomoses (Scholander, 1955; Steen & Steen, 1965; Midtgård, 1980). Some endotherms have large thermal windows that can function as heat radiators; for example, the large beaks of toco toucans (*Ramphastos toca*), southern yellow-billed hornbills (*T. leucomelas*) and southern ground-hornbill (*Bucorvus leadbeateri*) can increase blood flow and heat dissipation from their beaks via vasodilation (Tattersall *et al.,* 2009; Van De Ven *et al.,* 2016; Janse van Vuuren *et al.,* 2020). In bats, the highly vascularized and naked wing and tail membranes can act as thermal windows for heat dissipation at high *T*_e_ (Reeder & Cowles, 1951; Bartholomew *et al.,* 1970; Lancaster *et al.,* 1997). Some species, including fruit bats, can further lick their fur and wings in hot environments to promote evaporative cooling from these highly vascularized membranes (Welbergen *et al.,* 2008; Downs *et al.,* 2015). Given the complex roles that body size, morphological structures and appendages play in modulating heat exchange, more work is needed to include these traits in thermal risk assessments.

### Drinking behaviour

When exposed to high *T*_e_, animals require adequate access to water for evaporative cooling while maintaining a positive water economy to avoid deficits. Whether a species relies on drinking to maintain water reserves is typically defined by a species’ life history, but the extent of drinking dependence is shaped by abiotic (e.g. water availability, heat exposure) and biotic factors (e.g. diet, physiology, water economy; Conradie & Freeman, 2026). Drinking dependence may influence thermoregulatory performance. For instance, arid-zone birds in southern Africa that typically do not drink, even when water is available, have lower heat tolerance limits and evaporative cooling capacities compared to species that drink regularly (Czenze *et al.,* 2020*b*).

As *T*_e_ increases, the reliance on drinking water may increase, exposing species that are dependent on drinking to stronger sublethal trade-offs between thermoregulation, hydration and activity (Smit & McKechnie, 2015). For instance, non-drinking lark species are reported to select cooler microsites to reduce heat exposure risks compared to drinking counterparts, at the risk of lower foraging return and reduced body condition (Orolowitz *et al.,* 2023). In contrast, drinking lark species continue to forage in direct solar radiation, a behaviour that would otherwise be unsustainable under hot conditions if water was unavailable (Orolowitz *et al.,* 2023; A. R. Bourne personal Communication, 2019). Moreover, individuals typically not dependent on drinking or opportunistic drinkers may be forced to drink if evaporative cooling demands exceed metabolic water (derived from diet and/or metabolism) available for thermoregulation under extremely high *T*_e_. However, increased time spent drinking around water holes may expose species to additional thermoregulatory and predatory risks. Indeed, several mass mortality events have been reported around water holes (Towie, 2009), likely because of elevated hyperthermia risk due to the high metabolic demands of activity and solar heat loads reflected from water sources. While thermoregulation and exposure to thermal risks are tightly controlled by water consumption and availability, studies that explicitly contrast and examine the differences in risk between drinking and non-drinking species remain limited, particularly in bats.

### Roost or nesting ecology

Roosting behaviour and selection determine an individual’s thermal exposure, influencing both acute and chronic thermal risks. For nocturnal species, roost site selection is particularly important since individuals are confined to these sites during the hottest time of the day. Ground-nesting nocturnal birds, such as rufous-cheeked nightjars (*Caprimulgus rufigena*), can experience *T*_e_ of up to 60°C when roosting in fully sun-exposed microsites, during the hottest time of day (O’connor *et al.,* 2018). To allow for this roosting strategy, nightjars use gular flutter, a highly efficient mechanism to dissipate heat at high *T*_e_, while maintaining relatively low metabolic heat production (O’Connor *et al.,* 2017). Some bats also occupy hot roosts; for instance, the Angolan free-tailed bats (*Mops condylurus*) can experience *T*_e_ reaching 60°C and withstand these extremely high *T*_e_ values through a combination of facultative hyperthermia and evaporative cooling (Bronner *et al.,* 1999). Moreover, bats occupying hotter roosts (e.g. foliage-roosts) have higher heat tolerance limits and evaporative cooling capacity than those roosting in cooler sites (e.g. cave or cavity roosts; Noakes *et al.,* 2021; Czenze *et al.,* 2022*b*, 2024).

**Figure 2:**
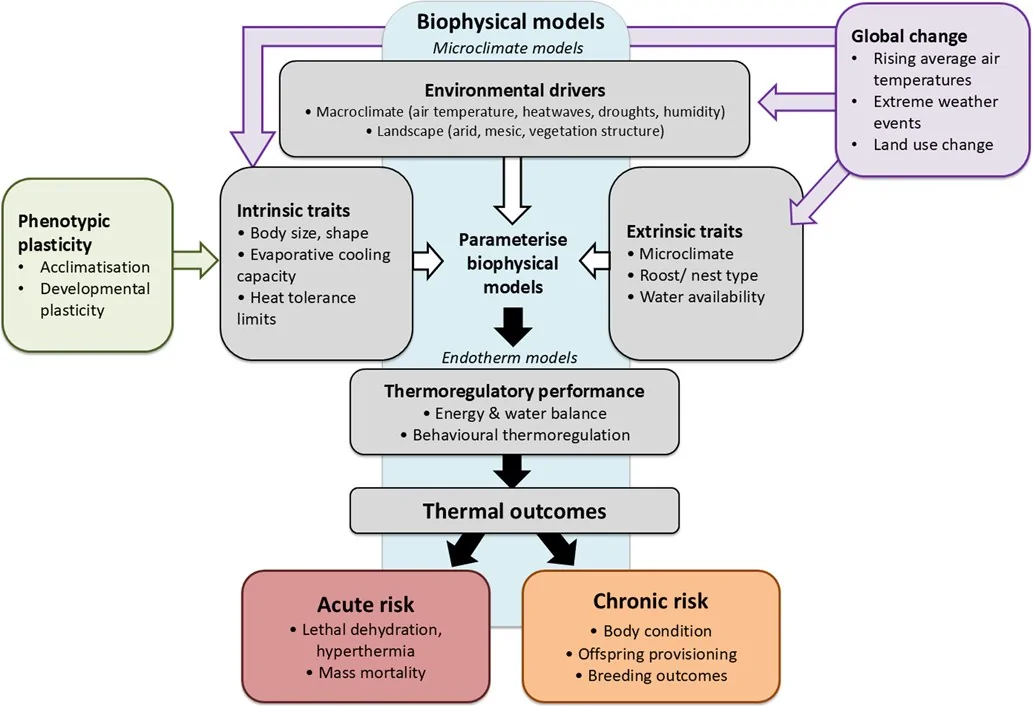
Conceptual framework linking global change, environmental drivers, species traits and phenotypic plasticity to acute and chronic thermal risks in birds and bats from a biophysical modelling perspective.

Despite these physiological adjustments, mass mortality events during extreme heat are disproportionately reported for species using exposed roosts, particularly foliage fruit bats, where limited insulation in their roost sites from *T*_e_ provides little buffer against lethal conditions (Welbergen *et al.,* 2008; Ratnayake *et al.,* 2019). This vulnerability is increasingly compounded by land-use change. In many regions, the loss of old-growth trees, natural cavities and intact forest structure is reducing the availability of thermally buffered natural roosts, forcing bats to rely more heavily on artificial structures (López-Baucells *et al.,* 2017). Such artificial structures, including bat boxes, often reach substantially higher *T*_e_ than natural roosts, increasing risks of lethal dehydration and hyperthermia (Mering & Chambers, 2014; Martin Bideguren *et al.,* 2019; Czenze *et al.,* 2022*b*). Integrating this variation in roost microclimates and roosting behaviour into biophysical models will be essential for accurately forecasting thermal risks across heterogeneous landscapes and for identifying species or populations most sensitive to future warming (Fig. 2).

## Adaptive responses

We refer to adaptive responses as variation in the physiology, behaviour and morphology of an individual or population that occurs via phenotypic plasticity or genetic adaptation to counteract an environmental stressor (Piersma & Drent, 2003; Angilletta *et al.,* 2010). Over recent decades, there have been increasing reports of adaptive responses of animals to climate change (reviews: Parmesan & Yohe, 2003; Parmesan, 2006; Thackeray *et al.,* 2016; Mainwaring *et al.,* 2017). Studies have typically focused on changes in phenology (e.g. timing of reproduction, migration and hibernation), range shifts (altitudinal and latitudinal) and morphology (e.g. body size and coat colour), with extensive reviews available for both birds (Crick, 2004; Fernando & Gunawardena, 2024) and bats (Sherwin *et al.,* 2013; Festa *et al.,* 2023; Kerth & Wolf, 2025). While there is some evidence of evolutionary responses to climate change in endotherms (Réale *et al.,* 2003; Stonehouse *et al.,* 2024), the pace of such responses relative to rapidly changing climates remains uncertain (Boyles *et al.,* 2011). Moreover, phenotypic plasticity is increasingly recognized as a critical mechanism by which individuals can respond to changing climates within their lifetime (Boutin & Lane, 2014; Charmantier & Gienapp, 2014; Sauve *et al.,* 2019).

Phenotypic plasticity refers to the ability of a genotype to express multiple phenotypes in response to environmental changes (Via *et al.,* 1995; Pigliucci, 2001; Piersma & Drent, 2003). It includes both phenotypic flexibility, reversible changes that can occur throughout an individual’s lifetime, and developmental plasticity, changes that become fixed on reaching maturity (Piersma & Drent, 2003; Kelly *et al.,* 2012). In the context of climate change responses, studies have focused on the plasticity of phenology and morphology (Canale & Henry, 2010; Bonamour *et al.,* 2019; Stollewerk *et al.,* 2025), with less attention to thermal physiology (Réale *et al.,* 2003; Boyles *et al.,* 2011). Understanding plasticity in thermoregulatory responses is crucial to accurately predict species’ responses to acute and chronic heat exposure, as these directly determine an individual’s ability to cope with high *T*_e_.

Phenotypic flexibility in thermal physiology occurs through acclimatization in response to changes in natural conditions or acclimation to laboratory conditions (Piersma & Drent, 2003; McKechnie, 2008). Seasonal acclimatization is well studied in endotherms, but due to the historical bias of research towards north-temperate regions, literature focuses on responses to cope with cold winter conditions (Lyman *et al.,* 1982; Dawson & Marsh, 1989; Swanson, 2010). Only relatively recently have studies investigated seasonal acclimatization in subtropical and tropical regions (McKechnie *et al.,* 2015; Pollock *et al.,* 2019), where summer maximum *T*_e_ and fluctuations in food availability may be more important environmental drivers than winter minimum *T*_e_ (Smit & McKechnie, 2010; Noakes *et al.,* 2016). A few studies have investigated seasonal acclimatization of the abilities of birds to cope with high *T*_e_ (e.g. Maloney & Dawson, 1994; Tieleman & Williams, 2002; Swanson, 2010; Cabello-Vergel *et al.,* 2024). For example, an arid-zone population of white-browed sparrow-weavers (*Plocepasser mahali*) had higher heat tolerance and evaporative cooling capacity during summer compared to winter, whereas there was no seasonal change in two populations of conspecifics from more mesic regions (Noakes *et al.,* 2016). A subsequent study found no interpopulation differences after acclimating sparrow-weavers to the same *T*_air_ treatments, with birds from all populations having higher heat tolerance in response to the hottest treatment (Noakes & McKechnie, 2019). We are only aware of one similar study on seasonal acclimatization in bats, where there was no seasonal change in maximum heat tolerance in four European vespertilionid species, but more gradual rates of increasing EWL with increasing *T*_air_ suggest enhanced water conservation during summer compared to spring (Czenze *et al.,* 2024).

While acclimatization in response to heat exposure under current climatic conditions yields insights into phenotypic flexibility, birds and bats are likely to experience novel environmental conditions in parts of their range due to climate change. Phenotypic flexibility is expressed through reaction norms, which are the range of phenotypic trait values a single genotype can produce in response to changing environmental conditions (Stone, 2000). These can be quantified using thermal acclimation experiments, but many studies only use two acclimation *T*_air_ treatments, giving little information on the shape of reaction norms (review: McKechnie, 2008). Research on avian basal metabolic rate suggests that reaction norms are approximately linear over acclimation *T*_air_ ranges (McKechnie *et al.,* 2007; Petit & Vézina, 2014), but with an upper and lower limit in the magnitude that basal metabolic rate can be adjusted (McKechnie, 2008; Noakes & McKechnie, 2020*a*). Less work has investigated acclimation in endothermic responses to cope with high *T*_air_, with a few studies on birds (e.g. Marder & Arieli, 1988; McKechnie & Wolf, 2004; Noakes & McKechnie, 2019) and, to the best of our knowledge, none on bats. Therefore, the shape and magnitude of reaction norms for heat tolerance and evaporative cooling capacity remain largely unknown (but see: Noakes & McKechnie, 2019).

Adding to the complexity is that free-ranging animals experience fluctuations in environmental variables, which may interact to drive phenotypic flexibility in thermoregulatory responses. Measuring responses over temporal scales in the wild can yield insights into reaction norms, with work on north-temperate birds reporting that metabolic heat production capacity and cold endurance are adjusted in response to fluctuations in minimum *T*_air_ over timescales of days to weeks during winter (Swanson & Olmstead, 1999; Petit & Vézina, 2014). In contrast, a study over several years in the Kalahari Desert of southern Africa found that metabolic responses of white-browed sparrow-weavers were adjusted in response to seasonal fluctuations in food availability rather than winter minimum *T*_air_ (Noakes & McKechnie, 2020*b*). This highlights that the environmental drivers of phenotypic flexibility may vary among species and climatic regions. We are aware of no comparable work on flexibility in heat tolerance and evaporative cooling capacity of free-ranging endotherms, but potential environmental drivers of adjustments include changes in maximum *T*_e_, humidity and water availability.

Developmental plasticity has received less attention, with the available literature suggesting that thermal challenges during development can either improve or constrain thermal tolerance later in life for birds, with no similar literature available for bats (review: Nord & Giroud, 2020). The embryonic period represents a particularly sensitive window in birds, during which prenatal thermal conditions shape the development of thermoregulatory control systems (Nichelmann, 2004). Studies on poultry report that exposure to heat during embryogenesis and the early postnatal period can lead to reduced physiological stress responses and improved thermal tolerance during subsequent heat exposure on reaching adulthood (Yahav & Hurwitz, 1996; Vinoth *et al.,* 2018; Ncho *et al.,* 2021). However, poultry studies are typically done in a production rather than an ecological context, with fewer studies investigating responses in wild-caught birds (Noakes & McKechnie, 2019; Nord & Giroud, 2020). In zebra finches, incubating parents produce a heat-call vocalisation at *T*_e_ > 26°C, which alters embryo development and affects thermal preferences at adulthood (Mariette & Buchanan, 2016; Mariette *et al.,* 2018). Individual finches with prenatal exposure to heat-call vocalizations appear to develop a heat-adapted phenotype as adults, including higher heat tolerance than conspecifics (Ton *et al.,* 2021; Pessato *et al.,* 2022). Studies on postnatal thermal effects on captive-bred Japanese quail show that not all thermoregulatory adjustments induced by early postnatal heat or cold exposure persist to adulthood after acclimating individuals to the same thermal conditions (Persson *et al.,* 2024, 2026). This highlights the need to distinguish between adjustments reflecting developmental changes and phenotypic flexibility (acclimation/acclimatization) in juvenile endotherms. A better understanding of the relative contributions of phenotypic flexibility and developmental plasticity to thermoregulatory responses in free-ranging birds and bats is critical for predicting how these taxa will respond to changing climates.

## Modelling avian and chiropteran thermal risks

Understanding and predicting how species respond to current and anticipated climatic changes is vital if we aim to conserve biodiversity. Thermal vulnerability assessments typically use either correlative or mechanistic approaches to predict the impacts of climate change (Erasmus *et al.,* 2002; Williams *et al.,* 2008; Foden *et al.,* 2013; Briscoe *et al.,* 2023). Correlative models are based on statistical, pattern-based techniques to classify species and areas at risk, using current distribution and climate data to identify areas of potential occupancy under future climates (Hilborn & Mangel, 1997; Erasmus *et al.,* 2002). Thus, the underlying processes driving these responses are left implicit (Hilborn & Mangel, 1997). While correlative models are useful for rapid, broad-scale assessments for multiple taxa, these approaches lack fine-scale details to untangle the mechanistic drivers of species vulnerabilities. In contrast, mechanistic approaches use process-based techniques to incorporate physiology and behaviour with climate data to predict how species will respond to changes in their environment (Mathewson *et al.,* 2017; Briscoe *et al.,* 2023). Here, we focus on a mechanistic approach that uses principles from biophysical ecology as the most promising and informative tool to predict acute and chronic thermal risks under current and future climates (Fig. 2; Briscoe *et al.,* 2023).

The field of biophysical ecology has a long-standing history; however, the application of biophysical models to understand and predict species responses to climate changes has only recently gained popularity (Briscoe *et al.,* 2023). Biophysical models are based on the conservation laws of thermodynamics to mathematically define the exchange of energy, mass and water of an animal with the surrounding environment, using the animal’s functional traits, physiology and behaviour (Porter & Gates, 1969; Bakken, 1976; Kearney & Porter, 2009). Thus, an organism’s energetic and hydric supply and demand can be quantified for any given environment and climate scenario (Briscoe *et al.,* 2023). The *NicheMapR* biophysical modelling package, based on the original work by Porter and colleagues (see Porter & Gates, 1969; Mccullough & Porter, 1971), has recently become freely available to biologists, further growing the popularity of the field (Briscoe *et al.,* 2023). This modelling package has been shown to accurately predict thermoregulatory responses (e.g. EWL, *T*_b_) measured in small birds under controlled dry (Conradie *et al.,* 2023) and humid air (Freeman and Conradie *et al.,* in prep), respectively. The accuracy of these predictions under laboratory conditions suggests that biophysical models may translate well into the field and provide the basis for modelling acute exposure to lethal dehydration and hyperthermia. For instance, biophysical models have been used to evaluate the role of thermoregulation in buffering species from the extremes of climate change (e.g. Kearney & Porter, 2009; Kearney *et al.,* 2016; Malishev *et al.,* 2018; de Mel *et al.,* 2025), how habitat loss and land-use change influence thermoregulation (e.g. Stark *et al.,* 2023; Wu *et al.,* 2024), and associated cost of increased cooling requirements (e.g. Riddell *et al.,* 2019, 2021).

Indeed, biophysical models have increasingly been used to understand, identify and predict free-ranging species’ acute vulnerability and resilience to climate change, with model outputs directly informing conservation and management action (e.g. Ma *et al.,* 2023; Strydom *et al.,* 2024; Zhang *et al.,* 2025). However, it is imperative to validate model outputs against responses measured in free-ranging individuals under natural conditions, and that the users have a firm understanding of the physiological and behavioural traits influencing species’ vulnerability before model outputs are used to inform conservation. Oversimplification of animal behaviour or environmental conditions experienced may lead to misleading results, widening the gap between reality and abstraction (Briscoe *et al.,* 2023; Conradie *et al.,* 2024). Collecting relevant biological data may be challenging due to permits, financial resources, species vulnerability status or the animals’ biology, but targeted data collection approaches (e.g. Conradie *et al.*, in press) or allometric adjustments from closely related species (e.g. Kearney *et al.,* 2016) may overcome these limitations. For example, the reliance of the enigmatic night parrot (100 g, *Pezoporus occidentalis*) of Australia on standing water has been modelled from related species under current and future conditions. The night parrot had its first confirmed sighting in 2013 after more than a century, and due to the rarity and elusive nature of this species, physiological data are difficult to obtain. Thus, allometrically adjusted data from the budgerigar (33 g) could be used to validate predictions of EWL, resting metabolic rate and *T*_b_ (Kearney *et al.,* 2016). Similarly, Zhang *et al.* (2025) demonstrated how biophysical models based solely on museum morphometrics and literature-derived estimates can be used to generate insight and hypotheses relevant to conservation efforts for Hainan gibbons (*Nomascus hainanus*), an endangered species lacking observational data. Moreover, most biophysical models currently assume that key physiological traits and thermal thresholds are fixed within species and across space and time, despite growing evidence that many traits exhibit substantial phenotypic plasticity (Boyles *et al.,* 2011). Ignoring plasticity may lead to overestimating thermal vulnerability where acclimatization or behavioural flexibility may provide buffering.

Biophysical models can also be used in a hypothetico-deductive framework to test ideas and lead to better implementation of action to outcome. By simulating physiological responses under different climate and land-use scenarios, biophysical models can predict potential outcomes before they are observed in wildlife (Zhang *et al.,* 2025). Moreover, predictions of energy and water demands can be integrated into other models, such as state-dependent models that explicitly predict behaviours resulting from trade-offs between thermal and hydric costs, foraging behaviour, predation risk, competition and social interactions (Sears *et al.,* 2016; Malishev *et al.,* 2018). Such approaches are particularly valuable for incorporating missed opportunity costs into thermal risk assessments, a regularly overlooked but crucial component of species-specific vulnerability (Cunningham *et al.,* 2021).

## Emerging questions

Our review sheds light on the acute and chronic heat-related risks birds and bats likely face, the key traits determining their vulnerability, and the promise of biophysical modelling as an informative tool to predict thermoregulatory costs. Together, these insights reveal a suite of emerging questions and methodological challenges that must be addressed to strengthen our understanding and predictive power if we aim to use biophysical models to inform conservation practices (Table 1).

**Table 1:** Emerging questions related to biophysical modelling of the acute and chronic thermal risks birds and bats face

**Theme**	**Emerging questions**	**Data needed**
Acute risks	Using biophysical models, can we predict at what point acclimatization is no longer sufficient or biologically realistic to buffer species from acute, lethal risks?	Physiology*:* reaction norms, environmental cues
Chronic risks	How do individual physiological thresholds and trade-offs translate into changes in population growth, range limits or extinction risk?	Life-history traits: behavioural observations, body condition, demography
Conservation planning	Can biophysical models identify general mechanistic rules that scale across taxa, climates and life-history strategies?	Behavioural: focal observations, camera traps, demographic monitoring
	Can we use behavioural data from ecologically similar species to parametrize acute and chronic thermal vulnerability models for species in which these data do not exist or are challenging to collect?	
	Can we test management interventions to buffer heat exposure (e.g. artificial shading)?	

An important knowledge gap highlighted by our review is the imbalance between quantitative assessments of the acute and chronic risks in bats compared to birds, with substantially fewer studies on sublethal fitness costs of heat exposure in bats. Key questions include how bats experience and mitigate heat exposure within daytime roosts, if night-time foraging returns are sufficient to offset daytime body mass loss due to evaporative demands following hot days and the extent to which bats can behaviourally escape unfavourable roost microclimates during extreme heat. Once these behavioural responses are better understood, research can turn towards understanding the circumstances where behavioural thermoregulation incurs sublethal fitness costs for bats. Addressing this taxonomic gap is critical if we aim to build generalisable frameworks of thermal vulnerability across endotherms, especially given bats’ ecological importance and increasing susceptibility to heat-related risks.

Beyond taxonomic gaps, comparative physiological limits remain poorly resolved across environmental gradients for birds and bats (but see Noakes *et al.,* 2016; Freeman *et al.,* 2022, 2024). While we suggest that humid environments appear to elevate risks of lethal hyperthermia and arid environments increase risks of lethal dehydration (Freeman *et al.,* 2022, 2024), the proximate causes of heat-related mortality likely vary among species and habitats in relation to a combination of variables. Similarly, a key emerging question is the extent to which current findings can be generalised globally, given the strong geographic bias of existing studies. Most studies examining the effects of heat and humidity on birds and bats have been conducted in a relatively small number of regions (e.g. southern Africa, Australia, North America) leaving large parts of the world poorly represented (e.g. Central and West Africa, East Asia). To understand how intra- and interspecific variation in dehydration tolerance limits and lethal hyperthermia thresholds may buffer species from heat-related risks, we need comparable data using standardized approaches. Establishing such standards is itself a major challenge, requiring careful consideration of methodological variables such as heating rates, exposure duration, humidity, starting temperatures and endpoints, all of which may influence estimated thermal tolerance limits. Comparative data across regions and taxa will allow us to identify patterns and thresholds in relation to different environmental factors, eventually allowing us to make broadscale predictions of acute vulnerability to heat exposure.

A further challenge is linking individual thermal vulnerability to population-level outcomes, which is essential given the potential for large-scale population crashes due to acute and chronic risks of heat exposure (Conradie *et al.,* 2019). To incorporate thermal risk into conservation management actions, we need more studies aimed at understanding how individual risks translate to population-level outcomes. In particular, we need to ask how thermoregulatory trade-offs and missed opportunity costs resulting in compromised body condition in individuals scale to reductions in survival, fecundity and recruitment in populations.

Although biophysical models provide an appealing framework for forecasting large-scale thermal risks, there is a concern that relying on them without adequate validation and testing may yield misleading results (Briscoe *et al.,* 2023). One approach is to ask whether models can reliably forecast mortality risk ahead of extreme heat events and test these predictions empirically. For instance, establishing long-term population monitoring programmes in areas predicted as high-risk heat-related mortality areas would allow model predictions of acute mortality risk to be tested directly. Here, research teams could intensify monitoring effort when extreme heat is forecast, quantify mortality rates or population changes before and after events and compare observed outcomes to model predictions. This prospective validation approach would represent a critical advance for conservation physiology, providing a rigorous test of model performance. In practice, however, long-term monitoring programmes are often constrained by funding limitations and bureaucratic processes. This highlights the importance of developing flexible monitoring approaches and the integration of existing citizen science and conservation monitoring networks to capture mortality and population responses.

Another way to improve the biological realism of biophysical modelling is to include physiological plasticity. While there is growing evidence that metabolic rate, heat tolerance and evaporative cooling capacity can shift through acclimatization or phenotypic plasticity (Noakes *et al.,* 2016; Czenze *et al.,* 2024), most biophysical models assume fixed trait values. To incorporate phenotypic plasticity into these models, several parameters must be defined. These include the magnitude and shape of reaction norms for heat tolerance and evaporative cooling capacity across environmental gradients, the timescales over which physiological adjustments occur, the environmental cues that trigger acclimatory responses and how these cues interact and the limits of plasticity beyond which buffering fails. While it is uncertain whether plasticity can sufficiently shift lethal thresholds to cope with anticipated changes in climate, quantifying the long-term consequences of developmental and adult exposure to elevated *T*_e_ and resolving trade-offs between plastic thermoregulatory adjustments and other fitness-related traits can shed light on the limits of plasticity. Addressing these questions will require coordinated efforts using controlled laboratory experiments to quantify reaction norms, field-based physiological measurements to study plasticity and associated trade-offs under natural environmental conditions and iterative biophysical modelling approaches to incorporate plasticity as a dynamic, mechanistic process in predictions of animal responses.

Finally, translating physiological and behavioural insights into conservation solutions remains an emerging priority. For example, what management interventions, such as artificial roost boxes, habitat shading, water provisioning or restoration of thermal refugia, most effectively reduce exposure to conditions associated with thermal risk? How can biophysical models be used to prioritize management actions by identifying when intervention is likely to yield meaningful population benefits? Addressing these applied questions is critical for ensuring that advances in conservation physiology translate into measurable outcomes for species persistence in a rapidly warming world.

## Conclusion

Our review provides mechanistic insight into the acute and chronic risks small endotherms experience due to climate change and the biophysical tools available to ecologists to predict these impacts on individuals and populations. Acute exposure can drive mortality during extreme heat, while chronic exposure to moderate-to-hot conditions can result in missed opportunities that influence population persistence. One common theme is that thermoregulatory capacity is influenced by environmental conditions and is likely a plastic trait, but the extent to which this trait is flexible remains in question. Biophysical models are an increasingly popular and powerful tool used to understand and predict the impacts of climate change. While these models are undoubtedly valuable, we emphasize the importance of understanding animal physiology and behaviour before interpreting the outputs of biophysical models for conservation planning and action. Moreover, we caution the oversimplification of biological and environmental processes used to parameterize biophysical models, which run the risk of misleading conservation efforts.
